# Supplementary material for: Identification of Freezing-Responsive microRNAs and Their Targets in Chinese Jujube by Small RNA and Degradome Sequencing
Source: Int J Mol Sci. 2026 Sep 4;27(17):7912. doi: 10.3390/ijms27177912 (PMC13566890; doi:10.3390/ijms27177912)
Supplement: Supplementary file 1 [file ijms-27-07912-s001.zip › Supplementary Material S2/novel_miR_141.pdf]

Provisional ID : novel_miR_141
Score total : 1514.8
Score for star read(s) : 3.9
Score for read counts : 1507.7
Score for mfe : 1.6
Score for randfold : 1.6
Score for cons. seed :
Total read count : 2969
Mature read count : 2829
Loop read count : 116
Star read count : 24

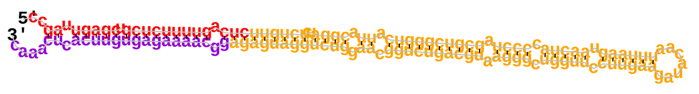

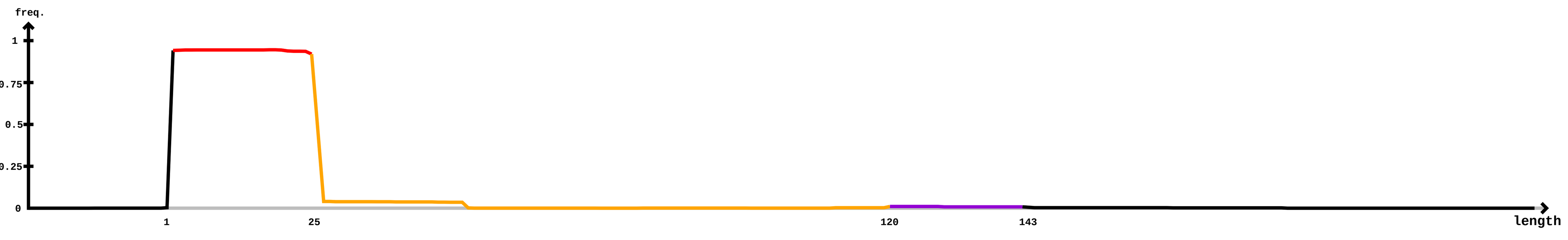


```
                                Mature                                                                                                   Star
5'- uuacuggauacuaagaucaaguccgauugagcugcucuuuugacucuugucucuaggcauuacugggcugcgaucccccaucaaugaauuuaacauagaaguuccuuggucgggaaugcagucuggcaaggucuggaugagaggcaaaagaguguucacucaaacuuuuucuuggcuuccaauaacaacauugagcguucaacaucuunnnnnnnnnnnnnnnnnnnnnnnnnnnnnnnnnnnnnnnnnnn -3'  obs
    uuacuggauacuaagaucaaguccgauugagcugcucuuuugacucuugucucuaggcauuacugggcugcgaucccccaucaaugaauuuaacauagaaguuccuuggucgggaaugcagucuggcaaggucuggaugagaggcaaaagaguguucacucaaacuuuuucuuggcuuccaauaacaacauugagcguucaacaucuunnnnnnnnnnnnnnnnnnnnnnnnnnnnnnnnnnnnnnnnnnn       exp
    ....((((..((((((..((((..((.(((((.(((((((((.((((((((..((((.((.((((((((((.((((.(((((.((((((......)))))).))))).)))).)))))))))).)).)))))))))))).)))))))))))))).))..))))..))))))..))))........((((....))))......  reads  mm  sample
    .........acuaagaucaaguccgauugagcugcuc...  1  0  LT-3
    .....................uccgauugagcugcucuuuugacuc...  1  0  LT-2
    .....................uccgauugagcugcucuuuugacuc...  2  0  LT-1
    .....................uccgauugagcugcucuuuugacuc...  5  0  LCK-3
    ......................ccgauugagcugcucuuu...  5  0  LCK-3
    ......................ccgauugagcugcucuuuu...  14  0  LCK-3
    ......................ccgauugagcugcucuuuu...  1  0  LT-1
    ......................ccgauugagcugcucuuuu...  1  0  LT-2
    ......................ccgauugagcugcucuuuug...  5  0  LCK-3
    ......................ccgauugagcugcucuuuugac...  2  0  LT-1
    ......................ccgauugagcugcucuuuugacu...  1  0  LT-1
    ......................ccgauugagcugcucuuuugacA...  1  1  LT-1
    ......................ccgauugagcugcucuuuugacu...  3  0  LT-2
    ......................ccgauugagcugcucuuuugacu...  1  0  JCK-3
    ......................ccgauugagcugcucuuuugacu...  5  0  LT-3
    ......................ccgauugagcugcucuuuugacu...  1  0  JCK-2
    ......................ccgauugagcugcucuuuugacu...  36  0  LCK-3
    ......................ccgauugagcugcucCuuugacuc...  1  1  LCK-3
    ......................ccgauugCgcugcucuuuugacuc...  1  1  LCK-3
    ......................ccgauugagcugcucuuuugacuc...  376  0  LCK-1
    ......................ccgauugagcugcucuuuugacuc...  6  0  JCK-1
    ......................ccgauugagcugcucuuuugacuc...  2  0  JT-3
    ......................ccgauugagcugcucuuuugCcuc...  1  1  LCK-3
    ......................ccgauugCgcugcucuuuugacuc...  1  1  JCK-2
    ......................ccgauugagcugcucuuuugacuc...  65  0  LT-3
    ......................ccgauugagcugcucuuuugacuc...  4  0  JT-1
    ......................ccgauugagcugcucuuuugacuc...  11  0  JCK-2
    ......................ccgauugagcugcucuuuugacuc...  1  0  JT-2
    ......................ccgauugagcugcucuuuugCcuc...  2  1  LCK-1
    ......................ccgauugagcugcucuuuugacuc...  1677  0  LCK-3
    ......................ccgauugagcugcucuuuugacuc...  13  0  JCK-3
    ......................ccgauNgagcugcucuuuugacuc...  1  1  LCK-1
    ......................ccgauugagcugcucuuuugacuc...  30  0  LT-2
```

```
                      Mature                                                                                                                Star
uuacuggauacuaagaucaaguccgauugagcugcucuuuugacucuugucucuaggcauuacugggcugcgauccccaucaaugaauuuaacauagaaguuccuuggucgggaaugcagucuggcaaggucuggaugagaggcaaaagaguguucacucaaacuuuuucuuggcuuccaauaacaacauugagcguucaacaucuunnnnnnnnnnnnnnnnnnnnnnnnnnnnnnnnnnnnnnnnnnnn
......................ccgauugagcugcucuuuugacuc.............................................................................................................................................................................................................  411  0  LCK-2
......................ccgauugaAcugcucuuuugacuc.............................................................................................................................................................................................................  1    1  LCK-2
......................ccgauugagcugcucuuuugCcuc.............................................................................................................................................................................................................  1    1  LT-1
......................ccgauugagcugcuNuuuugacuc.............................................................................................................................................................................................................  2    1  LCK-1
......................ccgauugCgcugcucuuuugacuc.............................................................................................................................................................................................................  1    1  LCK-2
......................ccgauugagcugcNcuuuugacuc.............................................................................................................................................................................................................  2    1  LCK-1
......................ccgauugagcugcucuuuugacuc.............................................................................................................................................................................................................  111  0  LT-1
......................ccgauugagcugcucuuCugacuc.............................................................................................................................................................................................................  1    1  LCK-2
......................ccgauugagcugUucuuuugacuc.............................................................................................................................................................................................................  7    1  LCK-3
......................ccgauugagcugcucuuuugacucA............................................................................................................................................................................................................  1    1  LT-2
......................ccgauugagcugcucuuuugacucA............................................................................................................................................................................................................  1    1  LT-3
......................ccgauugagcugcucuuuugacucA............................................................................................................................................................................................................  5    1  LCK-3
.......................cgauugagcugcucuuuugacuc.............................................................................................................................................................................................................  3    0  LCK-3
.......................cgauugagcugcucuuuugacuc.............................................................................................................................................................................................................  1    0  LT-1
........................gauugagcugcucuuuugacucuug..........................................................................................................................................................................................................  4    0  LCK-3
..........................uugagcugcucuuuugacu..............................................................................................................................................................................................................  1    0  LCK-3
.....................................uuuugacucuugucucuag...................................................................................................................................................................................................  1    0  LT-3
......................................uuugacucuugucucuaggca................................................................................................................................................................................................  3    0  LT-3
..............................................uugucucuaggcauuacugg.........................................................................................................................................................................................  4    0  LT-1
..............................................uugucucuaggcauuacugggc.......................................................................................................................................................................................  2    0  LT-1
..............................................uugucucuaggcauuacugggcugc....................................................................................................................................................................................  3    0  LT-3
..............................................uugucucuaggcauuacugggNugc....................................................................................................................................................................................  1    1  LT-2
..............................................uugucucuaggcauuacugggcugc....................................................................................................................................................................................  91   0  LCK-3
..............................................uuAucucuaggcauuacugggcugc....................................................................................................................................................................................  1    1  LCK-3
..............................................uugucucuaggcauuacugggcugc....................................................................................................................................................................................  6    0  LT-1
..............................................uugucucuaggcauuacugggcugcU...................................................................................................................................................................................  4    1  LCK-3
.......................................................................gauccccaucaaugaauuuaac..............................................................................................................................................................  1    0  LT-1
....................................................................................................uuccuuggucgggaaugc.....................................................................................................................................  2    0  LT-3
......................................................................................................................agucuggcaaggucuggaugagU..............................................................................................................  1    1  LT-1
....................................................................................................................................uggaugagaggcaaaaga.....................................................................................................  1    0  LT-1
....................................................................................................................................uggaugagaggcaaaaga.....................................................................................................  6    0  LCK-3
.............................................................................................................................................ggcaaaagaguguucacucaaac.......................................................................................  4    0  LT-2
.............................................................................................................................................ggcaaaagaguguucacucaaac.......................................................................................  15   0  LCK-3
.............................................................................................................................................ggcaaaagaguguucacucaaacA......................................................................................  4    1  LCK-3
.............................................................................................................................................ggcaaaagaguguucacucaaacA......................................................................................  1    1  LT-3
....................................................................................................................................................................uuuuucuuggcuuccaauaacaCc...............................................................  1    1  LT-1
....................................................................................................................................................................uuuuucuuggcuuccaauaacaac...............................................................  4    0  LCK-3
....................................................................................................................................................................uuuuucuuggcuuccaauaacaac...............................................................  4    0  LT-1
............................................................................................................................................................................................auugagcguucaacaucuu............................................  6    0  LT-2
```
